# Feasibility of Whole RNA Sequencing from Single-Cell mRNA Amplification

**DOI:** 10.1155/2013/724124

**Published:** 2013-12-23

**Authors:** Yunbo Xu, Hongliang Hu, Jie Zheng, Biaoru Li

**Affiliations:** ^1^Department of Computer Science, MCG, Augusta, GA 30912, USA; ^2^Renji Hospital of Shanghai, Jiaotong University School of Medicine, Shanghai, China; ^3^School of Computer Engineering, Nanyang Technological University, Singapore 639798; ^4^Department of Pediatrics, MCG, Augusta, GA 30912, USA

## Abstract

Single-cell sampling with RNA-seq analysis plays an important role in reference laboratory; cytogenomic diagnosis for specimens on glass-slides or rare cells in circulating blood for tumor and genetic diseases; measurement of sensitivity and specificity in tumor-tissue genomic analysis with mixed-cells; mechanism analysis of differentiation and proliferation of cancer stem cell for academic purpose. Our single- cell RNA-seq technique shows that fragments were 250–450 bp after fragmentation, amplification, and adapter addition. There were 11.6 million reads mapped in raw sequencing reads (19.6 million). The numbers of mapped genes, mapped transcripts, and mapped exons were 31,332, 41,210, and 85,786, respectively. All QC results demonstrated that RNA-seq techniques could be used for single-cell genomic performance. Analysis of the mapped genes showed that the number of genes mapped by RNA-seq (6767 genes) was much higher than that of differential display (288 libraries) among similar specimens which we have developed and published. The single-cell RNA-seq can detect gene splicing using different subtype TGF-beta analysis. The results from using Q-rtPCR tests demonstrated that sensitivity is 76% and specificity is 55% from single-cell RNA-seq technique with some gene expression missing (2/8 genes). However, it will be feasible to use RNA-seq techniques to contribute to genomic medicine at single-cell level.

## 1. Introduction

Clinical specimens are tremendously different from biological specimens in that the former contain mixed cells while the latter are mostly composed of pure cells. A mixed cell population in clinical samples can mask real results of genomic data, resulting in an inaccuracy of routine clinical genomic analysis and clinical genomic diagnosis. However, genomic medicine requires precise genomic profiling of clinical specimens to work for a clinical genomic diagnosis and to design personalized therapy for genetic and cancerous diseases. Like most routine diagnosis techniques [1, 2], clinical genomic analysis and genomic diagnosis techniques also have two prerequisites, that is, sensitivity and specificity, for clinical analysis and diagnosis [3–5]. In order to meet the requirements, two techniques can be considered: quantitative real-time PCR (Q-rtPCR) [6] and single-cell genomic analysis. After clinical genomic data, such as microarray data, is analyzed, Q-rtPCR is employed to support the microarray results by using similar primer design in the PCR as microarray probes [7]. Although Q-rtPCR is often used to confirm genomic data analysis as a standard test for genomics profile, the technique only selects a very small number of genes in the genomic profile. Moreover, most scientists only take genes of higher expression from the genomic data pool leading to only sensitivity measurements being demonstrated in genomic profile. To date, very few data demonstrate specificity from the genomic data pool. By contrast, single-cell genomic analysis can be applied for measurement of both sensitivity and specificity. Unfortunately, single-cell genomic techniques have different bottlenecks including a possibility of contamination of cells isolated from tissue samples and some comprehensive performance issues. Currently, most of the single-cell genomics are still only being used in reference laboratories and in some special fields such as specimens on glass-slides with local environmental changes (samples from department of pathology and genetics) [8] and sample of tumor tissue such as tumor infiltrating lymphocyte (TIL) and tumor cells [9]. Because TIL is easy to be cultured and very well identified from surface biomarkers (CD3, CD4, CD8, etc.), it is often used to develop single-cell genomic techniques. An example is the first single-cell genomic analysis model derived from the TIL [10].

TILs, one type of the cells located in tumor tissue, are responsible for immune surveillance to tumor cells [11]. If the TILs are in quiescent status, they lack spontaneous proliferation with a low metabolic rate. As the T-lymphocytes cause the loss of immune surveillance, these groups of cells attract interests of immunologists. Naturally, in native lymphocytes, quiescence reduces the resources (energy and size) to maintain a vast repertoire of T-cells. Only a small fraction of native lymphocytes will be clonally selected by antigen during the lifetime of the host. Moreover, some studies indicated that quiescence of CD8 T-cells is an actively maintained state rather than a defective state in the absence of the stimulated signals. Technically, we have successfully implemented a genomic approach at a single-cell level and implemented a modified differential display to analyze gene expression profiles of the CD8 T-cell in quiescent status obtained from human hepatic tumor tissue [12]. Based on the technology, we have uncovered several proteins involved in the regulation of T-cell quiescence including the lung-Krüpple-like factor (LKLF), which is a zinc finger-containing transcription factor that maintains T-cell quiescence [13]. Although the differential display technique can uncover some specific genes, it has limited routine applications for clinical specimens. For example, it will take several days to perform library processes of plasmid vectors with bacteria amplification followed by Sanger DNA sequencing to confirm them. Some laboratories also use RNA-microarray at the single-cell level [14]. More recently, a few studies attempt to apply single cell into the pipeline of RNA-seq [15]. However, analysis results of genomic profile are not clear at single-cell level. In order to develop a more applicable way to routinely work with single-cell genomics analysis and diagnosis of future genomic analysis in reference laboratories such as for personalized therapy, we study the feasibility of whole RNA genomic sequencing. We used the similar RNA specimens from differential display technique to run the RNA-seq. The goal of our study is to test if the RNA-seq technique can achieve similar results to our results of RNA differential display, thereby providing a more efficient platform for clinical genomic diagnosis.

## 2. Materials and Methods

### 2.1. Library Establishment

Single CD8 cells obtained from TIL of liver cancers were isolated, and a cDNA library was generated as previously reported [16]. Briefly, single CD8+ cells from TIL were directly lysed in an 8 *μ*L DNA digestion buffer with DNase I (Sigma). Two  *μ*L DNA digestion solution was added to a cocktail mixture containing 1 *μ*L dNTP, 1 *μ*L 50 mM 3′ anchor primer containing [5′-CTCTAAGCTT(T)_11_-3′], 2 *μ*L MgCl_2_, 1 *μ*L 10x buffer, 0.25 *μ*L RNasin, 0.25 *μ*L AMV reverse transcriptase, and 4.5 *μ*L sterile ddH_2_O (Promega, USA). First-strand synthesis was performed at 25°C 10 min, 42°C 1 hour, and 95°C 5 min. The cDNA was amplified by PCR with four arbitrary 5′ primers and oligo-T primers as in Table 1 in 25 *μ*L volume using AmpliTaq Gold from Perkin Elmer, USA. TIL CD8 cell library was stored at −80°C for further study. RNA of PBMN T-cell control (peripheral bold mononuclear cells) was isolated, and a cDNA library was generated similar to TIL.

### 2.2. RNA Whole Genomic Sequencing

*Sequencing Library.* The protocol is the same as shown in Illumina TruSeq RNA sampling process [17]. Briefly, after the DNA library stored at −80°C was fragmented with downstream end-repair process and a single “A” base addition, the fragment was ligated to adapters, purified by 2% agarose gel, and then enriched by PCR to create the final sequencing library. Finally, RNA single-end sequencing was performed using Solexa/Illumina Genome Analyzer II and using the standard protocol. The sequencing library was loaded to a single lane of an Illumina flow cell. The image was obtained using CASAVA 1.6 module to transfer BCL format into FASTQ format. Sequenced reads were generated by base calling using the Illumina standard pipeline.

*Alignment of Sequenced Reads. *The alignments were performed using the tool Galaxy. Galaxy was professionally developed for short oligonucleotide analysis, allowing up to 2 mismatches with the references. Sequenced reads were aligned to human transcript reference sequences from the human hg19 for the expression analysis at gene/transcript levels by Tophat and differential analysis by Cufflinks and Cuffdiff in Galaxy platform.

*Evaluation of Data.* To test the feasibility of sequencing, the correlation of gene expression between genes of RNA-seq whose data was from gene expression level as RPKM (reads per kilobase of transcript per million mapped reads) and single-cell differential display genomics (which we have published in 2009) [12] was used for RNA-seq gene expression in this study. FPKM (fragments per kilobase of exon per million fragments mapped) was used to study transcripts. In order to further analyze FPKM, we also used Bam ReadCount platform to analyze read count of splicing fragments.

### 2.3. RNA-Seq Data Analysis

To analyze the data of RNA-seq, the mapped genes were used to research the fold change by RPKM. Briefly, RPKM from PBMN and TIL were input into BRB ArrayTools (http://linus.nci.nih.gov/BRB-ArrayTools.html) [18]. We selected significance analysis of Microarray (SAM) with 1.2-fold change, false discovery rate 0.1, and permutation 100 to work on both RNA-seq profiles from PBMN and TIL.

### 2.4. Q-rtPCR to Confirm the Expression

The Q-rtPCR assay was performed in triplicate for each gene with the 25 *μ*L PCR reaction mixture, totaling at 50 uL containing 25 uL 2x SYBR Green (BioRad), 500 nM for each primer, RNA extracts, and iScript reverse transcriptase 1 uL. According to the primer conditions and manufacturer's recommendations, one step real-time PCR was 10 min at 50°C and 5 min at 95°C, followed by 45 cycles of denaturation for 10 s at 95°C and annealing/extension for 30 s at 55°C. The SYBR fluorescent signals were quantitatively analyzed as previously reported [12].

## 3. Results

### 3.1. Quality Control of RNA-Seq

**Figure 1:**
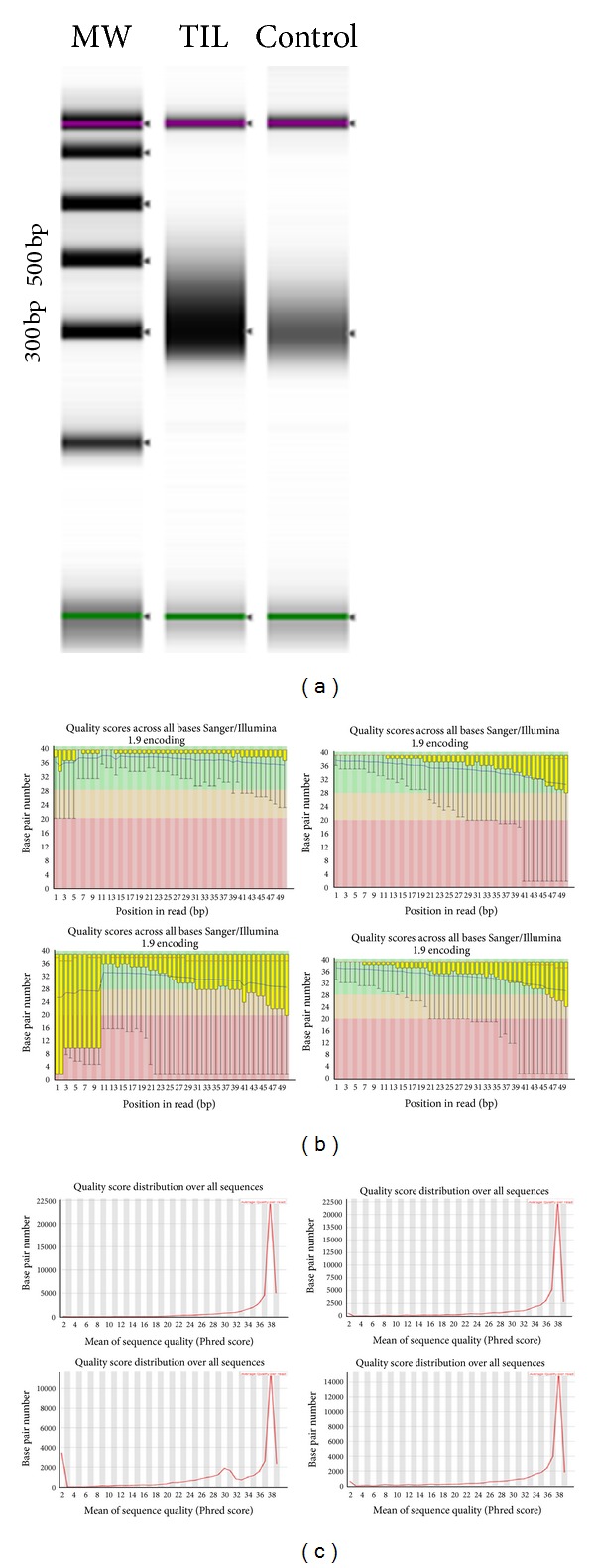
(a) Sequencing libraries were enriched by PCR and analyzed by 2100 bioanalyzer with 250–450 bp molecular weight. (b) and (c) Quality control for each base pair showed QC score >30.

After the library of DNA was fragmented with downstream end-repair process and a single “A” base addition, the fragments were ligated to adapters following Illumina TruSeq kit protocol and sequencing libraries were enriched by PCR and 2100 bioanalyzer as shown in Figure 1(a) with downstream purified under 2% agarose gel. RNA pair-end sequencing was performed using Solexa/Illumina Genome Analyzer II using the standard protocol. The sequencing library was loaded to a single lane of an Illumina flow cell. The image was performed using CASAVA 1.6 module to transfer FASTQ format. Sequenced reads and FSATQC were generated by base calling using the Illumina standard pipeline (Figures 1(b) and 1(c)).

**Figure 2:**
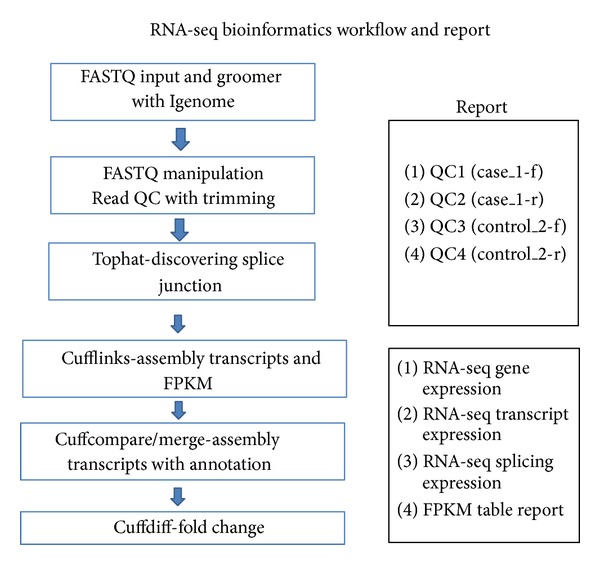
Bioinformatic analysis design for RNA-seq workflow and report.

**Figure 3:**
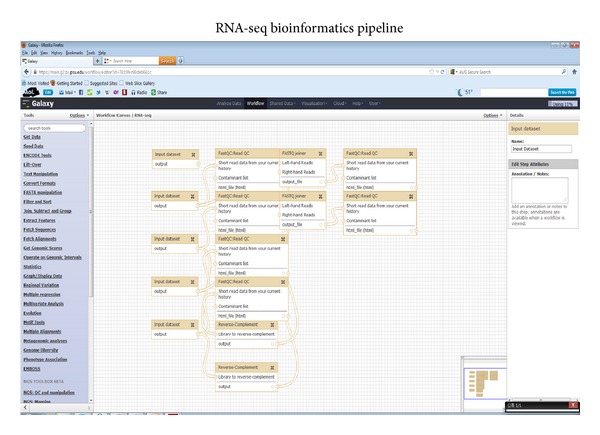
Bioinformatic analysis workflow from Galaxy analysis.

After the RNA-seq experiment harvested 19.6 million sequencing reads, 11.6 million aligned reads were achieved. All data analysis of the RAN-seq was performed in Galaxy local system as shown in Figure 2 and bioinformatics pipeline as shown in Figure 3. The numbers of mapped genes, mapped transcripts, or mapped exons were 31,332, 41,210, and 85,786 as Supplemental Tables 1, 2, 3, and 4, respectively, in Supplementary Material available online at http://dx.doi.org/10.1155/2013/724124.

### 3.2. Data Summary of RNA-Seq

**Figure 4:**
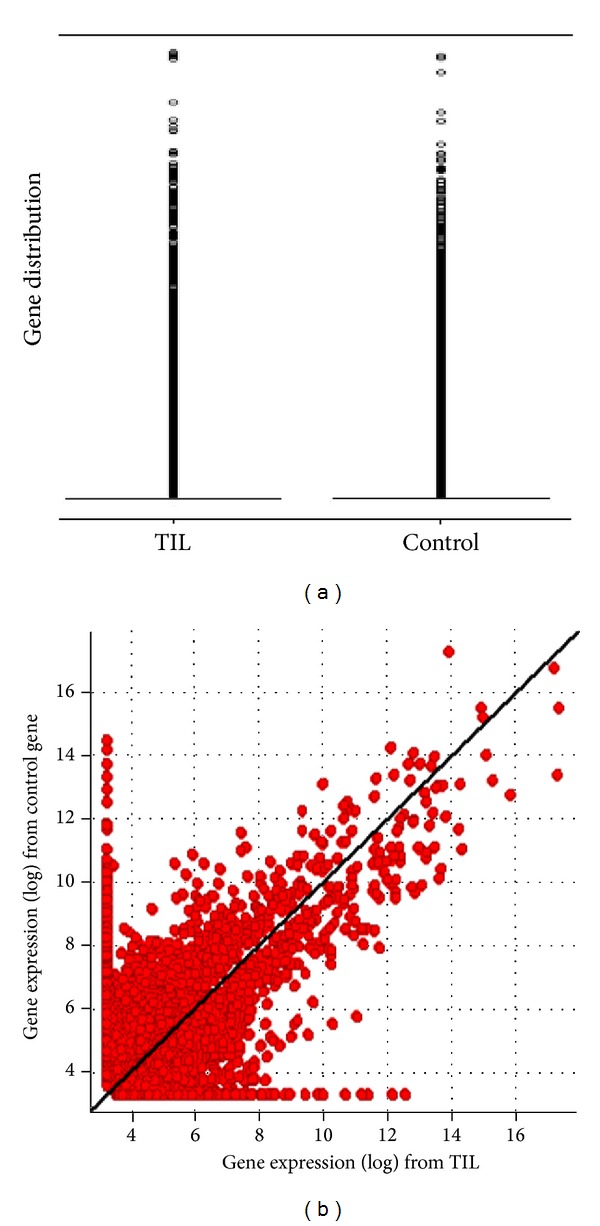
(a) Gene expression boxplot analysis for both TIL and control; (b) gene expression scatter-plot analysis for both TIL and control.

After mapping the genes, mapped transcripts or mapped exons were mined, and mapped genes were applied for data analysis. The results of the gene expression Boxplot are given in Figure 4(a). Correlation study was further confirmed by scatter plot analysis. Results of scatter-plot for both RNA-seq from TIL and PBMN were 0.65 as shown in Figure 4(b). SAM was used for gene expression mining. After SAM analysis, a total of 6767 genes passed filtering using the criteria of 0.1 FDR and 100 permutations. All fold changes are demonstrated in Supplemental Table 5.

### 3.3. Sensitivity and Specificity for RNA-Seq

After SAM analysis, a total of 6767 genes were filtered from SAM RNA-seq, and results were compared to 288 libraries from differential display. Eight silence genes were mined in single-cell differential display shown in Table 2, with 6 of 8 genes being mined using the RNA-seq technique. As with most single-cell genetics and genomics techniques, two of them (Sno-A and REST/NRSF) were still missed in RNA-seq results at single-cell level. In order to study measurement of sensitivity and specificity of RNA-seq, we selected 25 upregulated genes from TIL as positive genes and 11 downregulated genes as negative genes to analyze the measurement. After standard Q-rtPCR test, 19 out of 25 positive genes (Group-1) and 6 out of 11 negative genes (Group-2) were confirmed by standard Q-rtPCR test shown in Table 3. Although RNA-seq is considered a high-throughput technique, the sensitivity and specificity (76% and 55%, resp.) shown in Table 4 are all lower than those of differential display (100% and 86% which was published in Immunology, 2009) [12].

### 3.4. Splicing Discovery of Single-Cell RNA-Seq

In our previous experiment, TGF-beta had higher expression in TIL as measured by Q-rtPCR and differential display. Here, all family members of TGF-beta (TGF-beta1, TGF-beta2, and TGF-beta3) in TIL were expressed lower than those of T-cell in PBMN by single-cell RNA-seq as shown in Table 5. In order to address this question, we continue analyzing TGF-beta2 splicing as shown in Table 6. Surprisingly, TGF-beta2 RNA splicing from chr11 46392470 to 46393364 of TIL has a 3-fold change higher than those of PBMN. This result was further demonstrated by single-cell Q-rtPCR.

## 4. Discussion

A major task of clinical genomics is to study the levels of mRNA/protein expression and to discover functional SNPs related to a disease specific to the patient. Traditional approaches to identify and quantify genomic expression include mRNA microarrays [19], expressed sequence tags (EST) [20], serial analysis of gene expression (SAGE) [21], subtractive cloning for differential display (DD) [22] on mRNA, two-dimensional gel electrophoresis [23], mass spectrometry [24], protein microarray based antibody-binding for protein [25], single nucleotide polymorphism (SNP) microarray [26], and DNA-seq (whole genomics sequence and whole exome sequence) [27] for DNA. These traditional methods have been extensively utilized in the analysis of clinical specimens. Most specimens of animal and human tissue often contain multiple cell types with different gene expression profiles [28]. Results of clinical genomic profile will be unclear due to the multiple cell types at tissue level. Therefore, clinical genomics need to extend to a more precise technique and use data analysis procedures such as the special biospecimen process and special bioinformatics module and analysis. After a decade of effort, three fields have been quickly developed in clinical specimens for genomic analysis: (1) single-cell sampling with genomics analysis [29], (2) culture for a small number of cells (or single cells) with genomic analysis [30], and (3) different bioinformatics modules and applications with genomic analysis [31]. Single-cell sampling with genomic analysis plays an important role in all the three fields. For example, single-cell genomics are necessary in reference laboratory, specimens on glass-slides, and sample of tumor tissue such as TIL and tumor cells. Moreover, measurement of sensitivity and specificity at the single-cell level is an essential step to study genomic analysis in mixed-tissue level.

As we all know, the quantity of whole genome DNA is 6.6 pg with two copies in single cell [32]. Because of stable DNA with the mature downstream genomic DNA amplification technique, single-cell DNA genomic techniques have been successfully developed in SNP microarray and DNA-seq. Unfortunately, although the quantity of whole genome mRNA is approximately 1.0–30 pg (about 5 × 10^5^–1.5 ×10^6^ molecules based on different cell types) [33], unstable RNA will limit the development of single-cell RNA genomics techniques. The best way is to use a fresh cell lysate without purifying procedures to work on the technique [34]. To date, mRNA microarrays and differential display (DD) have been successfully applied for single-cell genomic analysis. Both have some pitfalls including missing genes and the possibility of contamination. The goal of our study is to study the feasibility of single-cell RNA-seq including measurement of sensitivity and specificity.

Results of the quality of RNA-seq demonstrated that most fragments ligated to adapters were 250–450 bp indicating an intact mRNA at single-cell level. Among the 19.6 million sequencing reads, 11.6 million reads were mapped. The numbers of mapped genes, mapped transcripts, and mapped exons were 31,332, 41,210, and 85,786. The QC results indicated that RNA-seq techniques can be used for single-cell genomic performance. After the mapped genes were applied for data analysis, the results of gene expression described with both boxplot and scatter-plot did not show bias. Unexpectedly, a total of 6767 genes were discovered in RNA-seq by SAM mining. The results suggest that RNA-seq is more powerful than differential display (only mining 288 libraries). The Q-rtPCR test demonstrated that sensitivity and specificity from RNA-seq technique were 76% and 55%, respectively. As most single-cell genomic techniques, gene missing rates are still higher (2/8 genes) including internal control analysis (2/6 genes) as shown in Supplemental Table 6. Encouragingly, RNA-seq at single-cell level is also able to uncover gene's splicing in mRNA expression as routine RNA-seq [35].

## 5. Conclusion

With this new RNA-seq technique, it would give researchers a new tool to study the single-cell genomics techniques. Results of RNA-seq including quality control, mapped reads, and the discovery rate demonstrated that RNA-seq techniques could be used for single-cell genomic analysis. The Q-rtPCR test demonstrated that sensitivity and specificity from RNA-seq techniques are lower than those from differential display with missing gene expression. This result demonstrated that RNA-seq still requires more time to be modified. However, it will be feasible to use RNA-seq techniques to contribute to genomic medicine at single-cell level.

## Supplementary Material

Table S1: FPKM table.
Table S2: Gene expression table.
Table S3: Transcript expression table.
Table S4: Splicing expression table.
Table S5: Gene analysis table.
Table S6: Internal control gene expression table.

## Figures and Tables

**Table 1:** Primer design.

Primer names	Sequences
(A) 5′-terminals	5′-CTCTGAATTCCTGATCCATG-3′
	5′-CTCTGAATTCCTTCATTGCC-3′
	5′-CTCTGAATTCCTGCTCTCAT-3′
	5′-CTCTGAATTCTCTGGAGGCA-3′
(B) 3′-terminals	5′-CTCTAAGCTT(T)_11_-3′

**Table 2:** Feasibility results of single-cell RNA-seq.

Genes	Single-cell DD	Single-cell RNA-seq
	Positive screening	RPKM
Total pool	288	6767
Tob	Yes	1.87048
Ski	Yes	1.20975
Sno-A	Yes	N/A
TGF-beta	Yes	Research
LKLF	Yes	0.42310
ERF	Yes	1.74318
REST/NRSF	Yes	N/A
c-Myc	Yes	1.19342

**Table 3:** Relationship between NGS-RPKM and quantitative rtPCR.

Group	Tracking_id	Gene_short_name	NGS-RPKM (fold)	Q-rtPCR (fold)
1	XLOC_000003	OR4G11P	2.082524442	4.12
1	XLOC_000004	OR4F5	1.912537997	2.54
1	XLOC_000018	WBP1LP6	2.098156562	0.62
1	XLOC_000019	CICP3	1.813002417	0.91
1	XLOC_000022	FAM87B	6.636153863	11.21
1	XLOC_000026	SAMD11	2.552233617	0.99
1	XLOC_000027	KLHL17	2.204370704	0.98
1	XLOC_000039	PUSL1	4.947080901	8.23
1	XLOC_000040	GLTPD1	2.846803765	3.32
1	XLOC_000041	TAS1R3	7.504951705	6.87
1	XLOC_000042	RP5-890O3.3	2.188584961	2.65
1	XLOC_001676	NDUFS2	10.64542674	15.21
1	XLOC_005590	RP11-57C13.5	3.13139258	2.12
1	XLOC_005591	PAPSS2	2.899004155	0.97
1	XLOC_005592	CFL1P1	2.49551064	0.87
1	XLOC_005593	PTEN	3.377478904	4.86
1	XLOC_014938	hsa-mir-3171	16.57512918	4.92
1	XLOC_014939	RP11-412H8.2	11.68354982	12.32
1	XLOC_014940	BTF3P2	8.902118851	7.23
1	XLOC_005113	APBB1IP	6.081246865	6.89
1	XLOC_005114	RNA5SP307	5.098972331	7.21
1	XLOC_022012	TOB1	1.870483205	2.12
1	XLOC_000060	SKI	1.209748635	2.43
1	XLOC_025300	ERF	1.743180444	3.12
1	XLOC_025768	MYC	1.193416773	2.17
2	XLOC_000028	PLEKHN1	0.316434457	1.12
2	XLOC_000029	ISG15	0.812291089	0.78
2	XLOC_000030	AGRN,RP11-54O7.14	0.020428549	0.45
2	XLOC_000031	RP11-465B22.3	0.031559673	0.86
2	XLOC_000654	MIR5584	0.120544436	0.92
2	XLOC_000655	C1orf228	0.284243365	1.13
2	XLOC_000656	KIF2C	0.387780493	1.23
2	XLOC_000657	RPS8,SNORD38A	0.3321431	0.89
2	XLOC_000658	SNORD46	0.293275977	2.21
2	XLOC_000028	PLEKHN1	0.316434457	0.92
2	XLOC_000029	ISG15	0.812291089	1.78

**Table 4:** Q-rtPCR test.

RNA-seq	Positive	Negative
24	19 (true positive)	5 (false negative)
12	6 (false negative)	6 (true negative)
36	Sensitivity (76%)	Specificity (55%)

**Table 5:** The results of TGF-beta.

TGF-beta	PBMN FPKM	TIL FPKM	Fold change
TGFB1	6.86176	2.32141	0.338311162
TGFB2	1.13462	1.12126	0.988225133
TGFB3	0.666142	0.103165	0.154869382

**Table 6:** The results of TGF-beta2.

TGFB	Chromosome	Splicing	Length (bp)	FPKM		Fold change	ReadCount		Fold change
				PBMN	TIL		PBMN	TIL	
TGFB2	chromosome 11	45944222–45945304	1082	0.99	0.85	0.86	11.83	10.12	0.86
TGFB2	chromosome 11	46164868–46165049	181	145.95	5.82	0.04	290.59	11.59	0.04
TGFB2	chromosome 11	46342256–46342968	712	30.07	0.95	0.03	235.50	7.42	0.03
TGFB2	chromosome 11	46392470–46393364	894	0.38	1.14	2.97	3.76	11.18	2.97
